# Influence of genetic variations in *TLR4 *and *TIRAP/Mal *on the course of sepsis and pneumonia and cytokine release: an observational study in three cohorts

**DOI:** 10.1186/cc9047

**Published:** 2010-06-03

**Authors:** Oliver Kumpf, Evangelos J Giamarellos-Bourboulis, Alexander Koch, Lutz Hamann, Maria Mouktaroudi, Djin-Ye Oh, Eicke Latz, Eva Lorenz, David A Schwartz, Bart Ferwerda, Christina Routsi, Chryssanthi Skalioti, Bart-Jan Kullberg, Jos WM van der Meer, Peter M Schlag, Mihai G Netea, Kai Zacharowski, Ralf R Schumann

**Affiliations:** 1 Department of Anesthesiology, Intensive Care Medicine and Pain Management, Hanse-Klinikum Stralsund, Große Parower Strasse 47-53, Stralsund 18435, Germany; 2 Department of Medicine, Radboud University Nijmegen Medical Center and Nijmegen Institute for Infection, Inflammation and Immunity (N4i), Geert Grooteplein 8, Nijmegen 6525 GA, The Netherlands; 3 4th Department of Internal Medicine University of Athens, Medical School, 1 Rimini, Athens 12462, Greece; 4 Clinic of Anesthesiology, Intensive Care Medicine and Pain Management, J.W.-Goethe-University Hospital, Theodor-Stern-Kai 7, Frankfurt am Main 60590, Germany; 5 Institute for Microbiology and Hygiene, Charite-University Medical Center Berlin, Dorotheenstrasse 96, Berlin 10117, Germany; 6 The Floating Hospital of Children, Tufts University, 755 Washington Street, Boston, MA 02111, USA; 7 Department of Medicine, University of Massachusetts Medical School, 55 Lake Ave North, Worcester, MA 01605, USA; 8 Institute of Innate Immunity, University of Bonn, Sigmund-Freud-Strasse 25, Bonn 53127, Germany; 9 Thurston Arthritis Research Center, University of North Carolina, 3330 Thurston Building, Chapel Hill, NC 27599, USA; 10 Center for Genes, Environment, and Health, National Jewish Health, 1400 Jackson Street, Denver, CO 80206, USA; 11 1st Department of Critical Care, University of Athens, Medical School, 45-47 Ipsilantou Street, Athens 10676, Greece; 12 Charite Comprehensive Cancer Center, Charite-University Medical Center Berlin, Invalidenstrasse 80, Berlin 10115, Germany

## Abstract

**Introduction:**

It has been proposed that individual genetic variation contributes to the course of severe infections and sepsis. Recent studies of single nucleotide polymorphisms (SNPs) within the endotoxin receptor and its signaling system showed an association with the risk of disease development. This study aims to examine the response associated with genetic variations of *TLR4*, the receptor for bacterial LPS, and a central intracellular signal transducer *(TIRAP/Mal) *on cytokine release and for susceptibility and course of severe hospital acquired infections in distinct patient populations.

**Methods:**

Three intensive care units in tertiary care university hospitals in Greece and Germany participated. 375 and 415 postoperative patients and 159 patients with ventilator associated pneumonia (VAP) were included. *TLR4 *and *TIRAP/Mal *polymorphisms in 375 general surgical patients were associated with risk of infection, clinical course and outcome. In two prospective studies, 415 patients following cardiac surgery and 159 patients with newly diagnosed VAP predominantly caused by Gram-negative bacteria were studied for cytokine levels in-vivo and after ex-vivo monocyte stimulation and clinical course.

**Results:**

Patients simultaneously carrying polymorphisms in *TIRAP/Mal *and *TLR4 *and patients homozygous for the *TIRAP/Mal *SNP had a significantly higher risk of severe infections after surgery (odds ratio (OR) 5.5; confidence interval (CI): 1.34 - 22.64; *P *= 0.02 and OR: 7.3; CI: 1.89 - 28.50; *P *< 0.01 respectively). Additionally we found significantly lower circulating cytokine levels in double-mutant individuals with ventilator associated pneumonia and reduced cytokine production in an ex-vivo monocyte stimulation assay, but this difference was not apparent in *TIRAP/Mal*-homozygous patients. In cardiac surgery patients without infection, the cytokine release profiles were not changed when comparing different genotypes.

**Conclusions:**

Carriers of mutations in sequential components of the *TLR *signaling system may have an increased risk for severe infections. Patients with this genotype showed a decrease in cytokine release when infected which was not apparent in patients with sterile inflammation following cardiac surgery.

## Introduction

Patients treated in ICUs following surgery or who are on ventilation support are prone to nosocomial infections [1,2]. The sequence of events leading to septic shock has been connected to the presence of biochemical products such as bacterial endotoxin or cytokines [3,4]. The innate immune system recognizes conserved microbial structures also termed pathogen-associated molecular patterns (PAMPs) by pattern recognition receptors (PRRs) [5]. Also, intrinsic mediators (danger/damage-associated molecular patterns (DAMPs)) can induce an inflammatory response involving similar host molecules [6]. Genetic variation of the pathogen recognition system is thought to explain, at least in part, individual differences in the reaction of patients to similar infectious stimuli. An influence of single nucleotide polymorphisms (SNPs) of pathogen recognition on susceptibility for infections and sepsis has therefore been suggested [7]. Toll-like receptors (TLRs) are one class of PRRs that sense bacterial, viral or fungal molecular structures or nucleic acids and induce systemic inflammation [8]. TLR4 recognizes lipopolysaccharide (LPS) of Gram-negative bacteria as well as intrinsic mediators such as high-mobility group box-1 (HMGB-1) or heat-shock proteins [9]. TLR-signaling involves at least four intracellular signaling adaptor molecules termed myeloid differentiation response factor 88 (MyD88), toll/interleukin-1 receptor (TIR)-associated protein (TIRAP), also known as MyD88-adaptor-like (Mal), toll-receptor-associated molecule (Tram) and toll-receptor-associated activator of interferon (Trif). TIRAP/Mal acts as a bridging adaptor recruiting MyD88 to TLR2 or TLR4 [10].

For *TLR4*, which is encoded on chromosome 9, several SNPs have been described, with the most frequent one being the Asp299Gly/Thr399Ile variation. There have been conflicting reports on the influence of this SNP on severity of infections or outcome in prospective trials [11].

Two SNPs within the gene coding for the intracellular signal transducer *TIRAP/Mal *(positioned on chromosome 11) have been recently described: one synonymous SNP (rs7932766) was shown to be associated with meningeal tuberculosis in Vietnamese patients [12]. Another *TIRAP/Mal *SNP (rs8177374) leading to an amino acid exchange (Ser180Leu) has been shown to protect from pneumococcal pneumonia when present in a heterozygous state [13]. The frequencies of both genetic variations in *TLR4 *and *TIRAP/Mal *have been recently studied worldwide in a comparative fashion, and it has been proposed that differences between regional populations can be attributed to selective pressure due to differences in sepsis susceptibility [14,15].

A direct cause and effect relation between cytokine release and carriage of SNPs of molecules implicated in response to stimulation with LPS is not easy to discern as a variety of factors such as the time of blood sampling and the intensity of the infectious stimulus may strongly influence the results. However, we attempted to perform an association between mortality in patients with sequential polymorphisms of the LPS receptor complex (*TLR4*-SNPs Asp299Gly/Thr399Ile and the *TIRAP/Mal*-SNP Ser180Leu) or patients homozygous for the *TIRAP/Mal *SNP in an observational retrospective cohort study of 375 patients. More precisely, we analyzed these genetic variations in different patient populations representing a large proportion of patients in ICUs for their ability to mount an adequate cytokine response, and furthermore investigated a potential influence for risk of and course of septic complications.

To further confirm our results in a group of 159 patients with ventilator associated pneumonia (VAP) we related clinical and cytokine data to the genotype. Additionally, in these patients monocytes were stimulated with LPS and cytokine release was correlated to the different genotypes. Finally, out of a third group of 415 patients following cardiac surgery matched pairs were used to determine whether non-infectious inflammatory signals would be influenced by the different genotypes.

## Materials and methods

### Patient inclusion and data collection

The studies were all approved by the local ethics committees of the respective institutions and DNA testing was permitted by either a signed broad written consent including DNA testing before surgery (Group I and Group III) or written informed consent provided by first-degree relatives in the case of patients with VAP (Group II). All steps were performed in accordance with the Helsinki declaration. Statistical analysis was carried out after anonymization of the patient's data. For all cohorts definition of sepsis (systemic inflammatory response syndrome (SIRS), sepsis, severe sepsis and septic shock) was based on published criteria [16]. In brief: sepsis was defined as the presence of criteria for SIRS in response to a documented or clinically suspected acute infection. Severe sepsis was defined as sepsis associated with either evidence of hypoperfusion with organ dysfunction or sepsis-induced hypotension. Septic shock was defined as sepsis with sepsis-induced hypotension requiring vasopressor therapy despite adequate fluid challenge along with the presence of hypoperfusion and organ dysfunction.

Patients in the first group (Group I) were all treated in the ICU of the Robert-Rössle-Klinik of the Charité-University Medical Center, Berlin, Germany between 1999 and 2004. Main inclusion criteria were a length of stay (LOS) of more than the mean LOS in the ICU (6.4 days) or death at any time following surgery. Both criteria were considered indicative of a complicated course. Medical records of these patients were examined for the development of infectious complications and accompanying medical conditions. Patients with end-stage tumor disease and chronic immunosuppression were excluded from the analysis. Out of 601 eligible patients, 375 fulfilled the inclusion criteria. Infections were defined as described by the National Institutes of Health clinical classification for nosocomial infections. Patients were followed up until discharge from the hospital. Prior to surgery, sampled blood or tissue specimens were examined for common *TLR4 *and *TIRAP/Mal *SNPs. The frequency of the *TIRAP/Mal *SNP in a subgroup of these patients has been reported recently [17].

Additionally two prospective studies including 159 patients with VAP (Group II) and 415 patients following cardiac surgery (Group III) were conducted. Patients in Group II were observed over the period of 2004 to 2006 in Athens, Greece. Clinical and serum cytokine data from a subgroup of 56 patients out of this cohort were included in previous studies and have been published elsewhere [18-20]. Patients were either hospitalized in the Department of Critical Care of the Evangelismos' General Hospital or in the second Department of Critical Care of the ATTIKON University Hospital of Athens, Greece. All patients were over 18 years of age and intubated for at least 48 hours before diagnosis of sepsis. Inclusion criteria were the concomitant presence of VAP, and sepsis, severe sepsis or septic shock. VAP was diagnosed if all of the following signs were present: a) core temperature above 38°C or below 36°C; b) new or persistent consolidation in lung X-ray; c) purulent trancheobronchial secretions; and clinical pulmonary infection score above six, as proposed elsewhere [1]. Exclusion criteria were the presence of a) neutropenia (< 500 neutrophils per mm^3^), b) HIV infection, and c) intake of corticoids (> 1 mg/kg of prednisone or equivalent for more than one month). Enrolled patients were followed-up for 28 days. For these patients the frequency of SNPs of *TLR4 *and *TIRAP/Mal *have already been reported [15].

Patients in Group III were part of a prospective cohort study determining the effect of genetic variations in innate immunity receptors on the cortisol response postoperatively. They were observed following elective cardiac surgery over the period 2005 to 2006 in the University Medical Center, Düsseldorf, Germany. Following written informed consent patients underwent cardiac or major vascular surgery, that is coronary artery bypass surgery, valve surgery or combined procedures employing extracorporal circulation. Exclusion criteria consisted of chronic corticosteroid medication and known disease in the hypothalamic-pituitary-adrenal-axis. Blood samples were obtained at five different time points: on the day of surgery between 07:00 and 09:00 am (0 hours) and on ICU admission (4 to 6 hours) and on the 1st to 3rd days following the procedure between 07:00 and 09:00 hours (24, 48 and 72 hours, respectively). We used a matched-control approach to reduce confounding factors of cytokine response. For each of the affected individuals, one patient from the wild type (*WT*)-group and the *TLR4 *group was chosen as a control. Therefore post-surgical cytokine levels were compared between patients with double mutations (n = 13) and patients with *Mal*-homozygous genotype (n = 5). A combination of 18 matched wild type patients and 18 *TLR4 *patients were chosen as controls resulting in a subgroup of 54 analyzed individuals. An additional group of 176 healthy blood donors with known age and gender who consented to anonymous genotyping served as controls for genotype frequency.

### DNA analysis

Tissue specimen and blood sampled earlier were examined with a previously described method [21]. *TLR4 *genotyping (rs4986790 for Asp299Gly, rs4986791 for Thr399Ile) was performed by restriction fraction length polymorphism- or melting curve analysis as described elsewhere [21,22]. Genotyping for *TIRAP/Mal *(rs8177374 for Ser180Leu) was achieved by melting curve analyses employing the Lightcycler 2.0 (Roche Diagnostics, Mannheim, Germany) using the following primers and probes: sense primer: GCCAGGCACTGAGCAGTAGT, antisense primer: GTGGGTAGGCAGCTCTTCTG, anchor probe; Red640-GATGGTGCAGCCC TCGGCCCC, sensor probe: AGGCCCAACAG CAGGG-FL. The melting peaks are at 53°C and 62°C for the wild type and mutated sequences, respectively. Due to secondary structures and allele biased amplification within the region of this SNP, analysis of heterozygous genotypes may sometimes result in false homozygous results. Therefore, all mutated samples were reanalysed by conventional restriction fraction length polymorphism as described in [13].

### Monocyte isolation and ex-vivo stimulation

Peripheral blood mononuclear cells were isolated after gradient centrifugation of heparinized whole blood over Ficoll Hypaque (Biochrom, Berlin, Germany) and three consecutive washings with PBS (pH 7.2) (Merck, Darmstadt, Germany); after flask incubation purity of adherent CD14-positive cells was more than 95%. Cells were stimulated with 1 ng/ml of purified endotoxin (LPS) from *Escherichia coli *O155:B5 (Sigma Co, St. Louis, MO, USA). TNF-α, IL-6 and IL-10 were estimated in supernatants [18].

### Measurement of cytokines

A 5 ml sample of blood was collected in a sterile and pyrogen-free tube. After centrifugation, serum was kept at -70°C until assayed. In Group II concentrations of TNF-α, IL-6 and IL-8 in serum, and of TNF-α, IL-6 and IL-10 in supernatants were estimated in duplicate by an ELISA (Diaclone, Paris, France). Lower detection limits were 3.12 pg/ml for TNF-α, 6.25 pg/ml for IL-6, 62.50 pg/ml for IL-8, and 12.50 pg/ml for IL-10. Concentrations of cytokines in supernatants were expressed as pg/10^4 ^cells. Cytokine analysis in patients of Group III was performed with the Cytokine Ten-Plex antibody bead kit (Biosource Europe, Nivelles, Belgium) on a Luminex xMAP system (Luminex, Austin, TX, USA) (Sensitivity for the assays: interferon (IFN)-γ: 5 pg/ml, IL-1b: 15 pg/ml, IL-2: 6 pg/ml, IL-4: 5 pg/ml, IL-5: 3 pg/ml, IL-6: 3 pg/ml, IL-8: 3 pg/ml, IL-10: 5 pg/ml, TNF-α : 10 pg/ml and granulocyte macrophage colony-stimulating factor: 15 pg/ml respectively). Data on cytokine values other than those presented in the current study are currently being analyzed for subsequent publication and are therefore not all included in this study.

### Statistical analysis

Differences in categorical data between patient groups were analyzed with the chi-squared test and with Fisher's exact two-tailed test for expected frequencies of less than five. Numerical data were expressed as means ± standard deviation (SD) if they followed a normal distribution or medians and interquartile range or median and 95% confidence intervals (CI) for non-normal distribution. For comparisons between groups the Kruskall-Wallis test, the Mann-Whitney U test or one-way analysis of variance with a Bonferroni correction and within a group the Wilcoxon's rank sum test were used, respectively. Odds ratios (OR) were determined by Mantel and Haenzel's statistics. For calculation, the SPSS for Windows software, release 14.0 (SPSS Inc., Chicago, IL, USA) and the Prism 5.01 for Windows (GraphPad Software, San Diego, CA, USA) software were used. A two-tailed *P *< 0.05 was considered significant.

## Results

### Frequency of TIRAP/Mal and TLR4 polymorphisms

In all patients examined (n = 949), 252 carried the *TIRAP/Mal *SNP with 229 being heterozygous and 23 homozygous for this allele. The resulting allele frequency was 0.145, which is consistent with other reports and our own control group consisting of 176 healthy individuals from Germany (Table 1). In all patient cohorts, this SNP was in Hardy-Weinberg Equilibrium. Of 127 individuals with *TLR4 *variants two patients displayed the Thr399Ile allele only, and three displayed only the Asp299Gly allele. As recently described for European populations in all other patients, the Asp299Gly and Thr399Ile SNPs were cosegregating [15]. Three patients were homozygous for both alleles. The allele frequency for any *TLR4 *SNP was 0.069, which is in line with previous studies [11].

**Table 1:** Distribution of genotypes in the patients and healthy controls

	Genotype			*TIRAP/Mal*		
			Wild type	heterozygous	Homozygous	

**Group I** **(n = 375)**		Wild type	240 (64.0%)	75 (20.0%)	10 (2.7%)	AF TIRAP/Mal: **0.140**
	***TLR4***	heterozygous	41 (10.9%)	7 (1.9%)	1 (0.3%)	
		homozygous	--	1 (0.3%)	--	
	AF TLR4: **0.068**					

			Wild type	heterozygous	Homozygous	

**Group II** **(n = 159)**		Wild type	106 (66.7%)	40 (25.2)	1 (0.6 %)	AF TIRAP/Mal: **0.142**
	***TLR4***	heterozygous	9 (5.7%)	3 (1.9%)	--	
		homozygous	--	--	--	
	AF TLR4: **0.038**					

			Wild type	heterozygous	Homozygous	

**Group III** **(n = 415)**		Wild type	254 (61.2%)	89 (21.4%)	7 (1.7%)	AF TIRAP/Mal: **0.151**
	***TLR4***	heterozygous	45 (10.8%)	14 (3.4%)	4 (1.0%)	
		homozygous	2 (0.5%)	--	--	
	AF TLR4: **0.080**					

			Wild type	heterozygous	Homozygous	

**Controls (n = 176)**		Wild type	122 (69.3%)	26 (14.8%)	2 (1.1%)	AF TIRAP/Mal: **0.102**
	***TLR4***	heterozygous	20 (11.4%)	3 (1.7%)	1 (0.6%)	
		homozygous	1 (0.6%)	1 (0.6%)	--	
	AF TLR4: **0.080**					

Allele frequencies equal between the groups. All groups in Hardy-Weinberg-Equilibrium: For TLR4: Group 1: *P *= 0.55; Group II: *P *= 0.62; Group III: *P *= 0.64; Controls: *P *= 0.36. For TIRAP/Mal: Group 1: *P *= 0.12; Group II: *P *= 0.40; Group III: *P *= 0.54; Controls: *P *= 0.34.

AF, allelic frequency, Mal, MyD88-adaptor-like, MyD88, myeloid differentiation response factor 88, TIR, toll/interleukin-1 receptor, TIRAP, TIR-associated protein, TLR, toll-like receptor.

Overall, 30 individuals had a combination of *TIRAP/Mal *and *TLR4 *SNPs. One patient was *TLR4 *homozygous and *TIRAP/Mal *heterozygous. Of 29 *TLR4 *heterozygous mutation carriers, 24 were *TIRAP/Mal *heterozygous and 5 homozygous. The distribution of SNPs in the studied patients and the cohort of healthy controls is shown in detail in Table 1.

### Clinical influence of genotypes on postoperative infection severity in surgical patients

The 375 patients enrolled in the first cohort of patients (Group I) were unrelated European Caucasians. The mean age of patients was 61.8 years (SD: ± 12.6) and 137 (36.5%) patients were female. Overall, 203 patients in Group I developed infections. No association with single genotypes and susceptibility for infection or specific microorganisms was found. For risk associations with severe sepsis we compared the SNP carriers (41 heterozygous *TLR4*, and 10 homozygous and 75 heterozygous carriers of the *TIRAP/Mal*-SNP) with *WT*-patients (n = 240). In our analysis, the *TIRAP/Mal *homozygous genotype influenced patient morbidity resulting in higher risk of severe infections (OR: 7.3; 95% CI: 1.89 to 28.50; *P *< 0.01). Furthermore, in nine patients the combination of *TLR4 *and *TIRAP/Mal *SNPs significantly contributed to the risk of severe infections as shown in Table 2 (OR 5.5; 95% CI: 1.34 to 22.64; *P *= 0.02). This effect was not influenced by the type of infection in these two genotype groups. However, an influence of infection type was observed in the remaining subgroups (*TLR4*, *TIRAP/Mal *heterozygous and wild type-patients). In these patients presence of pneumonia and peritonitis contributed to the risk of severe infections. A detailed summary of this patient cohort is presented in the supplementary material in Tables S1, S2 and S3 in Additional file 1.

**Table 2:** Association between septic complications and genotype in 375 patients of Group I

Genotype	Severe Infections (n = 102)	^a^OR	95% CI	*P *value
**Wild-type** **(n = 240)**	64 (26.7%)	0.87	0.51 - 1.48	0.69
Mutant alleles				

***TLR4 *(n = 41)**	10 (24. 4%)	1.57	0.73 to 3.37	0.33
***TIRAP *(het)** **(n = 75)**	15 (20.0%)	0.87	0.49 to 1.56	0.65
***TIRAP *(hom) (n = 10)**	7 (70.0%)	**7.33**	1.89 to 28.50	**< 0.01**
***TIRAP/TLR4 *genotype (n = 9)**	6 (66.7%)	**5.50**	1.34 to 22.64	**0.02**

*^a^*OR (odds ratio) when comparing severe infections (severe sepsis and septic shock) to patients with no infection. Calculated by comparison of w*ild type*-patients with patients bearing other variants through Mantel-Haenzel's statistic and Fisher's exact test.

CI, confidence interval, het, heterozygous, hom, homozygous, Mal, MyD88-adaptor-like, MyD88, myeloid differentiation response factor 88, OR, odds ratio, TIR, toll/interleukin-1 receptor, TIRAP, TIR-associated protein, TLR, toll-like receptor.

### Cytokine release and monocyte stimulation in patients with ventilator-associated pneumonia

To further study the apparent impact of these double mutations on patients in ICUs we examined 159 Caucasian patients of Greek ethnicity (Group II) as part of a prospective cohort study. All these patients were on ventilator support as part of the treatment for brain hemorrhage, multiple injuries, primary respiratory failure or postoperative support, and developed VAP predominately caused by Gram-negative bacteria during their treatment. Mean age of patients was 59.6 years (SD: ± 18.6). Forty (25%) patients were female. Patient characteristics were similarly distributed over the genotype groups as shown in Tables S1, S2 and S4 in Additional file 1. Of the patients, 106 were carriers of only *WT *alleles; 9 were carriers of only *TLR4 *SNP alleles; 41 were carriers of at least one *TIRAP/Mal *SNP allele; and 3 were carriers of both *TLR4 *and *TIRAP/Mal *SNP alleles. Septic shock occurred among 47 (44.3%), 6 (66.7%), 19 (46.3%), and none of them, respectively.

**Figure 1:**
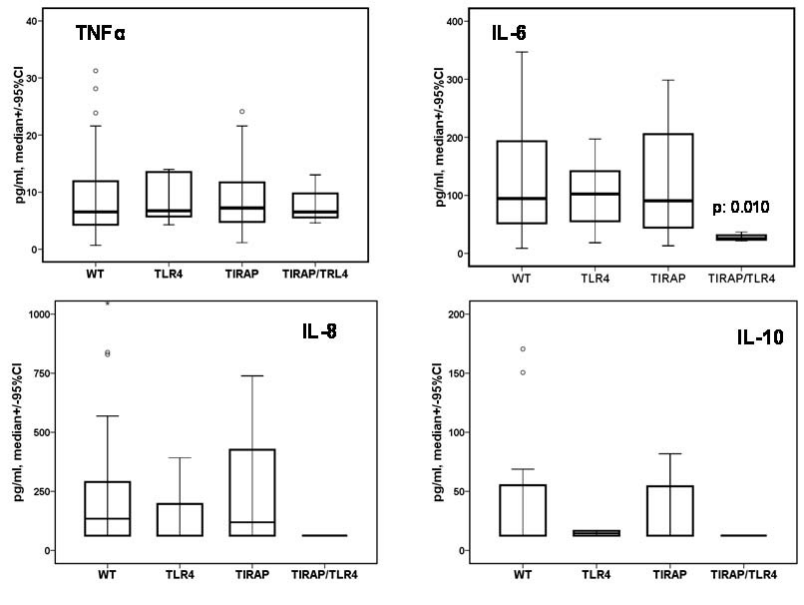
**Impact of *TIRAP/Mal *or *TLR4 *polymorphisms or their combinations on circulating cytokine levels**. Cytokine serum levels (pg/ml) were measured on day 1 after diagnosis of ventilator-associated pneumonia in Group II. Values are shown as median +/- 95% confidence interval (CI) and as mean +/- standard error. Number of patients in each group: Controls = 106, *TIRAP/Mal *(heterozygotes and homozygotes reported together) = 41, *TIRAP/Mal-TLR4 *= 3, *TLR4 *= 9. *P *values refer to significant differences compared with patients bearing the wild-type for all tested polymorphisms. Comparison calculated with Mann-U Whitney test. In this figure *TIRAP/Mal *is named *TIRAP *for readability.

When comparing circulating cytokine levels and their correlation to the *TLR4 *and *TIRAP/Mal *genotype, individuals with combined mutations in *TLR4 *and *TIRAP/Mal *had very low circulating cytokine levels of IL-6 (*WT*-patients vs. *TIRAP/Mal *+*TLR4*, *P *= 0.01 for IL-6) and IL-8, while all other individuals, including those with single mutations in either *TLR4 *or *TIRAP/Mal *had elevated cytokine levels at day 1 after diagnosis of pneumonia. Figure 1 shows cytokine values in patients of Group II on day 1 after diagnosis of VAP.

**Figure 2:**
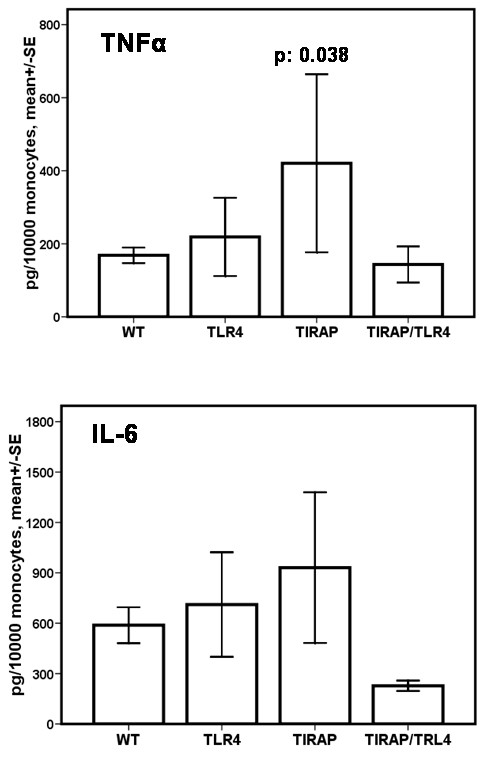
**Impact of *TIRAP/Mal *or *TLR4 *polymorphisms or their combinations on monocyte release of IL-6 and TNF-α following LPS-stimulation**. Monocytes were isolated from patients on day 1 after diagnosis of ventilator-associated pneumonia. Cells were then stimulated *in vitro *with lipopolysaccharide (LPS) for 24 hours, and IL-6 content was assessed by ELISA as described in the Materials and Methods section. Shown are mean values ± standard deviation. Patient numbers are as described in Figure 1. SE, standard error; WT, wild type.

To investigate the influence of different genotypes on the cytokine induction pattern, monocytes from these patients were isolated and stimulated *ex-vivo *with LPS. Concentrations of TNF-α and IL-6 in cell supernatants of patients bearing the wild-type phenotype and of carriers of *TIRAP/Mal *or *TLR4 *polymorphisms showed an increase of cytokine levels. Individuals with a double mutation in *TLR4 *and *TIRAP/Mal*, however, exhibited a lack of inducibility for TNF-α and IL-6 on day 1 after diagnosis (Figure 2). Patients carrying no mutations or either *TLR4 *or *TIRAP/Mal *mutations showed a stronger induction of IL-6 following LPS-stimulation compared with patients with double mutations, although the difference did not reach statistical significance. Similar results were seen for TNF-α although less pronounced. No differences in cytokine concentrations of monocyte supernatants between patients bearing the wild-type and carriers of polymorphisms were found on day 7 (data not shown).

### Influence of genotypes on cytokine release following cardiac surgery

**Figure 3:**
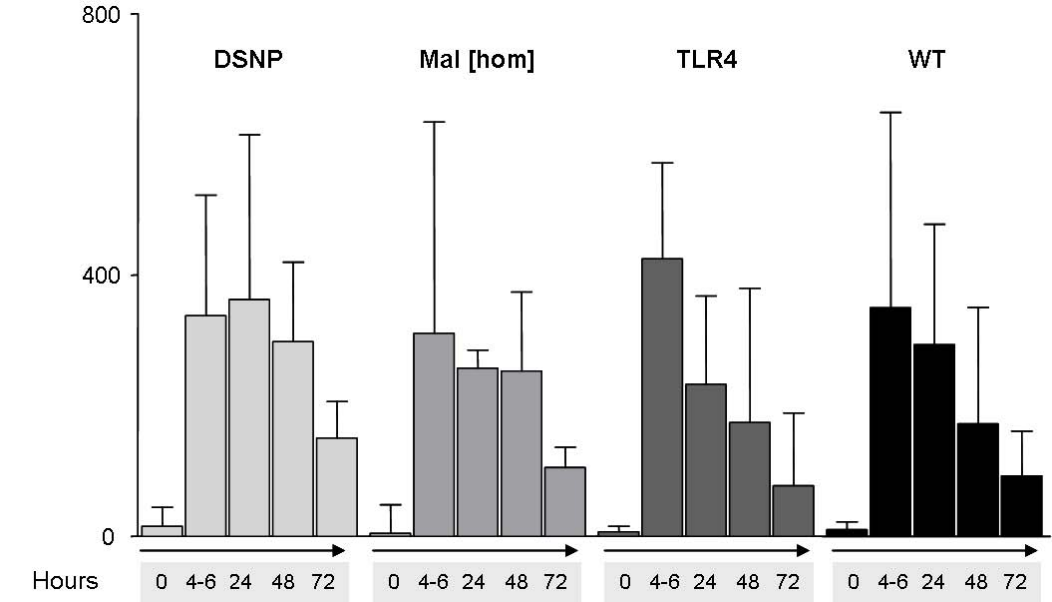
**Time-dependent cytokine release in cardiac surgery patients**. Timepoints were defined as: preoperative (0), immediately postoperative (4 to 6 hours), first postoperative day (24 hours), 2nd (48 hours) and 3rd postoperative day (72 hours). Samples were taken between 7:00 and 9:00 a.m. except postoperatively. Different genotype groups (DSNP = *Mal/TLR4 *combination, light grey bars; *Mal[hom] *= *TIRAP/Mal*-homozygous, grey bars; *TLR4 *= patients with TLR4-SNPs, dark grey bars; *WT *= wild-type patients, black bars). There were no statistical differences between the genotype groups at the timepoints. All values are shown as median and interquartile range.

A third group of patients was then examined to distinguish between a predominately sterile inflammatory stimulation as compared with the stimulus towards immune cells by bacterial ligands. The patients studied following cardiac surgery were all of Caucasian descent. Patient characteristics of this cohort and the matched patients are shown in Tables S1, S2 and S5 in Additional file 1. There were no statistically significant differences in the cytokine levels following surgery over the study period between the subgroups (Figure 3). The postoperative course was not related to cytokine levels in these patients (data not shown). Interestingly, patients receiving cardiac surgery exhibited markedly higher levels of IL-6 following the procedure in all studied genotypes as compared with patients suffering from infection approximately 24 hours following the insult. Although the results were obtained by different cytokine-detection assays there seem to be different mechanisms responsible for the release of cytokines in operated patients.

## Discussion

Patients suffering from infections seem to react individually to a similar insult. This capability to combat an infection is thought to be at least in part influenced by genetic factors [23]. Despite important advances in the understanding of the pathophysiological processes leading to sepsis and septic shock [4,24,25], knowledge on the role of genetic factors contributing to sepsis susceptibility has not yet translated into improved outcome [26,27].

In the first part of this study we were able to show an association between the risk of severe infections and a combination of genetic variants in sequential molecules of the LPS-sensor consisting of *TLR4 *and its adaptor *TIRAP/Mal*. The presence of *TLR4 *mutations in combination with *TIRAP/Mal *variants - either homozygous or heterozygous - resulted in a statistically significant increase in the risk of severe infections. Despite the fact that the number of patients carrying these mutations is low, we found intriguingly low serum levels of pro-inflammatory cytokines in double-mutant individuals in a second cohort (Group II). Additionally we found that monocytes of these patients show decreased cytokine production upon stimulation with LPS. One might speculate that moderate defects in TLR4 and TIRAP/Mal function may accumulate to induce significant alterations of TLR4 dependent signals. However, clinical outcome data in this cohort could not support the findings with regard to sepsis severity. One reason for this discrepancy could be that the second cohort consisted of more severely ill patients already suffering from infections caused by highly resistant Gram-negative pathogens. Moreover, other confounding factors may influence those effects such as preexisting conditions, type of infection in surgical patients or causing microrganisms. As the innate immune response to bacterial infection has to be mounted early and effectively, genetic influence on cytokine response in infection may determine effectiveness of bacterial killing [28].

Supporting our results, it has been recently found that severe sepsis and septic shock is associated with decreased expression of TLR4 on host immune cells [29]. Thus, a lack in TLR4 signaling may be associated with a worse outcome of disease, which also correlates with the recent findings suggesting that immunosuppression caused by negative regulators of TLR signaling are associated with sepsis mortality [30].

To further differentiate whether the observed lack in inducibility of cytokines depended on the type of inflammation, either *bacterial *infection or *sterile *inflammatory stimulus, we also assessed postoperative cytokine response following cardiac surgery (Group III). This strong inflammatory reaction is a consequence of ischemia-reperfusion injury and is observed frequently following procedures involving cardiopulmonary bypass [31]. DAMPs are thought to be involved in this process and previous studies have associated this phenomenon to the innate immune system [32]. In this non-infectious group, we were not able to show a difference in the cytokine response between the genotype groups. This could be in part explained by the hypothesis that the involvement of endogenous danger signals compared with bacterial ligands involves further elements of the innate immune system, or that TLR4 is not a main receptor of DAMPs in this group of patients. This could potentially explain the difference observed in cytokine concentrations between patients following cardiac procedures as compared with the patient group with pneumonia.

TIRAP/Mal is an important adaptor molecule for intracellular signaling of both TLR4 and TLR2 [10]. As one of four adaptors for TLR4 signaling [33], TIRAP/Mal functions as a 'bridging molecule' for MyD88 [34]. A recently published study postulated a protective effect of the heterozygous *TIRAP/Mal *variant (Ser180Leu) for pneumococcal disease [13]. A study on patients with severe forms of tuberculosis, the rate of meningeal manifestations was associated with a synonymous *TIRAP/Mal *SNP, but not with the above mentioned variant [12]. Both investigations found an altered cytokine release in cell-stimulation assays, supporting the view that these SNPs are functionally relevant. Therefore, a second important finding of our study was the significantly increased risk for severe infections in *TIRAP/Mal*-homozygous patients. This supports previous findings of Khor and colleagues [13]. Although we could not observe a significant reduction in risk for *TIRAP/Mal*-heterozygous patients compared with *WT*-patients as seen in the Khor and colleagues study, comparison of homozygous and heterozygous patients was statistically different. As only one patient in Group II was *TIRAP/Mal*-homozygous, no comparison of patients was possible here.

Activation of TLR4 may lead to a differential use of intracellular adaptors depending on the ligand that is bound to it [35]. Thus, a disturbance in the TLR4-TIRAP/Mal axis could lead to a predominant activation of the TIRAP/Mal-independent signaling pathway, which could explain the reduced release of nucleur factor (NF)-kB-dependent cytokines. Therefore, a potential 'shunting' of signals via a second pathway (Trif/Tram) could result in an unbalanced cytokine release brought about by interferon regulatory factor 3 (IRF3) and the release of type I interferon-α and -β [36]. It has recently been shown that IRF3 is crucial for endotoxin tolerance and activation may result in a reduction of cytokine release upon LPS-stimulation [36]. It is not known whether this may lead to a change in the clinical course of sepsis, but animal models showed an influence on sepsis mortality if IRF3 was pharmacologically inhibited [37]. In contrast, in *TIRAP/Mal *knock-out mice or in macrophages with nonfunctional *TIRAP/Mal*, the cytokine release via NF-κB was strongly reduced, while IRF3-dependent signals were almost unaltered [33]. A clinical trial such as the one presented here can only yield associations of genetic variations and the observed findings. For proving a causal link an animal model is needed with transgenic mice carrying either the human gene(s) of interest or its mutated variant(s). Our interpretation that the decreased ability to induce cytokines is a cause of an altered course of infectious diseases at this point is pure speculation.

An interesting observation was the lack of differences of cytokine stimulations between patients carrying *WT *alleles and those carrying SNP alleles on day 7. The only probable explanation may come from the known changes of responsiveness of monocytes to *ex vivo *stimulation during the course of sepsis, which could depend on other factors such as secondary infections or the anti-inflammatory response. We were not able to associate these mechanisms to clinical or cytokine data in our patients.

In addition, recently generated data in *TLR2 *and *TLR4 *knock-out mice give strong evidence that the TLR-pathway plays a pivotal role in the stress-hormone axis after LPS-challenge as well [38]. So the course of infections in patients with the described SNPs is potentially linked to an altered stress response and may therefore influence severity of sepsis. However, data on the influence of *TIRAP/Mal *variants on the stress-hormone axis are lacking to date.

The *TLR4 *SNP Asp299Gly/Thr399Ile studied here was found to cosegregate in 98% of the individuals in these European populations, confirming previous data in the literature [11]. In the present study the 299/399 *TLR4 *haplotype, when present without *TIRAP/Mal *mutations, was only weakly associated with susceptibility and course of disease in both groups. Our results also failed to show an association of the *TLR4 *299/399 haplotype with the incidence or type of microorganisms in surgical infections. Small previous studies on the *TLR4 *Asp299Gly/Thr399Ile haplotype showed higher disease susceptibility and higher incidence of infections caused by Gram-negative microorganisms [39], but this was not supported by subsequent studies [40,41]. The normal responses of individuals bearing this allele following LPS challenge *in vitro *[42-44] and *in vivo *[45] support this lack of association. Whether the presence of *TLR4 *haplotypes containing only the Asp299Gly or Thr399Ile SNPs is associated with Gram-negative infection susceptibility cannot be concluded from our study, due to the small number of patients carrying these haplotypes. However, in individuals bearing the Asp299Gly *TLR4 *haplotype alone an altered cytokine response to LPS and increased susceptibility for sepsis has been reported [15,46].

As mentioned above, both *TLR4 *and *TIRAP/Mal *genetic variants differ significantly in their frequency according to geographic locations [14,15]. This suggests that selective pressure has been present as a consequence of different disease susceptibilities. In these studies differences in cytokine release according to the genetic variations have been proposed to be the key functional factor supporting the results presented here.

## Conclusions

Recognition of microbial products via TLRs and subsequent signaling is crucial for the innate immune system to initiate a response. Genetic alterations affect this response and are related to individual variations in the course of sepsis. In summary, our studies describe a novel association between common genetic polymorphisms in sequential elements of the endotoxin recognition system (*TLR4 *and the intracellular signaling adaptor *TIRAP/Mal*) and the course of sepsis and pneumonia. However, we were not able to show an effect on susceptibility to infections. This could indicate that variant genes in the innate immune receptor system apparently are not affecting the capability to sense invading microorganisms, but rather the appropriate initiation and modulation of the innate immune response. These findings are supported by the fact that following cardiac surgery a strong and non-infectious stimulus does not lead to an altered cytokine response when comparing the genotype groups. Further clinical and experimental studies are necessary to elucidate the role of combined genetic variations in complex diseases such as sepsis.

## Key messages

• Individuals carrying genetic variations in both, *TLR4 *and the TLR signal transducer *TIRAP/Mal *had a higher risk of developing severe infectious complications following surgery as shown in two large studies including a total of 790 patients.

• Individuals carrying these two genetic variations had significantly lower cytokine levels both, in serum and following *ex-vivo *monocyte stimulation.

• These differences were not observed in a non-infectious patient cohort with post-surgical SIRS indicating the effects observed to be microorganism-driven.

• We conclude that the increased risk for developing septic complications of double SNP carriers may be caused by an impaired ability to react to pathogens with an inflammatory response.

• Genotyping for innate immune receptors may identify individuals with increased risk for septic complications who should be subject to intensified prophylactic measures.

## Abbreviations

CI: confidence interval; DAMP: danger/damage associated molecular patterns; ELISA: enzyme-linked immunosorbent assay; GM-CSF: granulocyte macrophage colony-stimulating factor; HMGB-1: high-mobility group box-1; IFN-γ: interferon-γ; IL: interleukin; IRF3: interferon regulatory factor 3; LOS: length of stay; LPS: lipopolysaccharide; Mal: MyD88-adaptor-like; MyD88: myeloid differentiation response factor 88; NF-κB: nuclear factor-κB; OR: odds ratio; PAMP: pathogen-associated molecular pattern; PBS: phosphate buffered saline; PRR: pattern recognition receptor; SD: standard deviation; SIRS: systemic inflammatory response syndrome; SNP: single nucleotide polymorphism; TIR: toll/interleukin-1 receptor; TIRAP: TIR-associated protein; TLR: toll-like receptor; TNF-α: tumor necrosis factor-α; Tram: toll-receptor-associated molecule; Trif: toll-receptor associated activator of interferon; VAP: ventilator-associated pneumonia; WT: wild type.

## Competing interests

The authors declare that they have no competing interests.

## Authors' contributions

OK and ELatz performed the data collection in the surgical patient group. EJG-B, MM, CR and CS performed the data collection and cytokine stimulation experiments in patients with VAP. AK and KZ performed data collection and cytokine measurements in cardiac surgery patients. DYO recruited control patients and performed data collection. LH and ELorenz performed the genotyping. PMS, DAS, ELatz, MGN, BK, JMWvdM and KZ contributed to conception and design of the study. Statistical analysis was performed by OK and EJG-B. RRS headed the project, supervised and conducted the study. OK, EJG-B, AK and RRS wrote the manuscript with input from all other authors.

## Supplementary Material

Additional file 1 **Supplementary Tables S1 to S5**. The supplementary tables contain detailed information about the studied cohorts. Click here for file
