# Supplementary material for: Influence of genetic variations in TLR4 and TIRAP/Mal on the course of sepsis and pneumonia and cytokine release: an observational study in three cohorts
Source: Crit Care. 2010 Jun 3;14(3):R103. doi: 10.1186/cc9047 (PMC2911747; doi:10.1186/cc9047)
Supplement: Additional file 1 — Supplementary Tables S1 to S5. The supplementary tables contain detailed information about the studied cohorts. [file cc9047-S1.doc]

**Influence of genetic variations in TLR4 and TIRAP/Mal**

**On the course of sepsis and pneumonia and cytokine release:**

**An observational study in three cohorts**

*Oliver Kumpf, MD; *Evangelos J. Giamarellos-Bourboulis, MD, PhD; *Alexander Koch, MD; Lutz Hamann, PhD; Maria Mouktaroudi, MD; Djin-Ye Oh, MD; Eicke Latz, MD, PhD; Eva Lorenz, MD, PhD; David A. Schwartz, MD, PhD; Bart Ferwerda, MD; Christina Routsi, MD, PhD; Chryssanthi Skalioti, MD; Bart-Jan Kullberg, MD, PhD; Jos W. M. van der Meer, MD, PhD; Peter M. Schlag, MD, PhD; Mihai G. Netea, MD, PhD; Kai Zacharowski, MD, PhD, FRCA and Ralf R. Schumann, MD, PhD

**Supplemental material**

**Table S1** Reasons for ICU admissions in the different patient cohorts

| ***Type of surgery / Reason for admission [No (%) of patients]*** | |
| --- | --- |
| ***Group I (n=375)*** |  |
| *Upper-GI resection* | 123 (27.6) |
| *Colorectal resection* | 105 (30.4) |
| *^a^Other abdominal* | 78 (22.1) |
| *Thoracic* | 27 (7.7) |
| *Limb and others* | 42 (12.5) |
| ***Group II (n=159)*** |  |
| *Brain hemorrhage* | 26 (43.9) |
| *Multiple injuries* | 26 (29.3) |
| *Respiratory failure* | 68 (14.6) |
| *Postoperative support* | 27 (2.4) |
| *Other* | 12 (9.8) |
| ***Group III (n=415)*** |  |
| *CABG* | 242 (58.1) |
| *Valve repair* | 41 (9.9) |
| *Combined* | 54 (13.0) |
| *^b^Other* | 78 (18.8) |
| *ECC* | 375 (90.4) |

*^a^*Other abdominal operations: pancreas-, liver- and multiple visceral organ resection. *^b^*Other procedures: Aortic replacement, peripheral vascular procedure or thoracic procedure. CABG = Coronary artery bypass grafting, ECC = extracorporal circulation, GI = gastro-intestinal

**Table S2 Characteristics of the different patient groups:**

| ***Characteristic*** | ***Group I***  ***(n=375)*** | ***Group II***  ***(n=159)*** | ***Group III***  ***(n=415)*** | ***Controls***  ***(n=176)*** |
| --- | --- | --- | --- | --- |
| *Age, (years, mean ± SD)* | 61.8 ± 12.6 | 59.6 ± 18.6 | 66.5 ± 12.2 | 30.8 ± 3.7 |
| Male/female | 238 / 137 | 119 / 40 | 305 / 110 | 152 / 24 |
| ***Co-existing diseases [No (%) of patients]*** | |  |  |  |
| Arterial hypertension | 157 (41.9) | 16 (10.1) | 279 (67.2) | - - |
| Myocardial disease | 129 (34.4) | 31 (19.5) | 401 (96.6) | - - |
| Diabetes | 77 (20.5) | 23 (14.5) | 132 (31.8) | - - |
| Lung pathology | 69 (18.4) | 3 (1.9) | 41 (9.9) | - - |
| Renal pathology | 25 (6.7) | 2 (1.3) | 57 (13.7) | - - |
|  |  |  |  |  |

SD = standard deviation

**Table S3.** Detailed clinical characteristics of 375 postoperative patients (Group I):

|  | | ***Wild-type Control (n=240)*** | | ***Any mutant TLR4***  ***(n=41)*** | | ***Homozygous mutant TIRAP (n=10)*** | | ***Heterozygous mutant TIRAP (n=75)*** | | ***Any mutant TIRAP/TLR4 (n=9)*** | |
| --- | --- | --- | --- | --- | --- | --- | --- | --- | --- | --- | --- |
| *Age (years, mean ± SD)* | | 61.4 ± 13.5 | | 64.0 ± 10.6 | | 58.5 ± 15.0 | | 61.7 ± 10.6 | | 68.6 ± 7.4 | |
| *Male/female* | | 154/86 | | 18/23 | | 7/3 | | 54/21 | | 5/4 | |
| *ASA* | | 2.8 ± 0.7 | | 2.8 ± 0.8 | | 2.6 ± 0.7 | | 2.8 ± 0.6 | | 3.1 ± 0.3 | |
| ***Co-existing diseases [No (%) of patients]*** | | | |  | |  | |  | |  | |
| *Arterial hypertension* | | 100 (41.7) | | 19 (46.3) | | 3 (30.0) | | 29 (38.7) | | 6 (66.6) | |
| *Myocardial disease* | | 89 (37.1) | | 11 (26.8) | | 1 (10.0) | | 25 (33.3) | | 3 (33.3) | |
| *Diabetes* | | 40 (16.7) | | 11 (26.8) | | 3 (30.0) | | 19 (25.3) | | 4 (44.4) | |
| *Lung pathology* | | 51 (21.3) | | 4 (9.8) | | 1 (10.0) | | 8 (13.3) | | 5 (55.5) | |
| *Renal pathology* | | 15 (6.3) | | 3 (7.3) | | 1 (10.0) | | 5 (6.7) | | 1 (11.1) | |
| ***Type of surgery / [No (%) of patients]*** | | | |  | |  | |  | |  | |
| *^a^Upper-GI resection* | | 72 (30.0) | | 18 (43.9) | | 2 (20.0) | | 27 (36.0) | | 4 (44.4) | |
| *Colorectal resection* | | 67 (27.9) | | 12 (29.3) | | 3 (30.0) | | 21 (28.0) | | 2 (22.2) | |
| *^b^Other abdominal* | | 53 (22.2) | | 6 (14.6) | | 4 (40.0) | | 13 (15.3) | | 2 (22.2) | |
| *Thoracic* | | 19 (7.9) | | 1 (2.4) | | 1 (10.0) | | 6 (8.0) | | - - | |
| *Limb and others* | | 29 (12.1) | | 4 (9.8) | | - - | | 8 (10.7) | | 1 (11.1) | |
|  | | | |  | |  | |  | |  | |
| ***Infections / [No (%)]*** | **(n=135)** | | **(n=18)** | | **(n=9)** | | **(n=34)** | | **(n=7)** | |  |
| *Pneumonia* | | 54 (40.0) | | 7 (38.9) | | 4 (44.4) | | 17 (50.0) | | 2 (25.0) | |
| *Peritonitis* | | 22 (16.3) | | 4 (22.2) | | 3 (33.3) | | 6 (17.6) | | 4 (50.0) | |
| *Abscess* | | 40 (29.6) | | 5 (27.8) | | 1 (11.1) | | 8 (23.5) | | - - | |
| *^c^Other* | | 19 (14.1) | | 2 (11.1) | | 1 (11.1) | | 3 (8.8) | | 1 (12.5) | |

*^a^*Upper-GI resection: Esophageal resection, Gastrectomy. *^b^*Other abdominal surgery: pancreas-, liver- and multiple visceral organ resection. Percentage of infections relates to the number of total infections in a genotype group.  *^c^*Other infections: Urinary tract, catheter related, skin/soft tissue. ASA = American Society of Anesthesiologists, GI = gastro-intestinal, SD = standard deviation

**Table S4.** Detailed clinical characteristics of 159 patients with ventilator-associated pneumonia (VAP) and sepsis (Group II)

|  | ***Wild-type Control (n=106)*** | ***Any mutant TLR4 (n=9)*** | ***Heterozygous TIRAP (n=40)*** | ***Any mutant TIRAP/TLR4 (n=3)*** |
| --- | --- | --- | --- | --- |
| *Age (years, mean ± SD)* | 60.7 ± 18.4 | 62.9 ± 18.3 | 56.2 ± 19.4 | 57.0 ± 19.9 |
| *Male/female* | 80/26 | 7/2 | 29/12 | 3/0 |
| *CPIS (mean ± SD)* | 7.68 ± 1.08 | 8.00 ± 1.19 | 7.75 ± 1.48 | 8.75 ± 1.70 |
| *SAPS II score (mean ± SD)* | 39.0 ± 14.4 | 38.4 ± 12.4 | 38.4 ± 13.6 | 28.0 ± 4.0 |
| *White blood cells (/nl, mean ± SD)* | 13.6 ± 7.3 | 11.5 ± 4.2 | 12.7 ± 4.9 | 15.3 ± 4.6 |
| *^a^pO_2_/FiO_2_ (mean ± SD)* | 228.7 ± 121.1 | 223.9 ± 82.7 | 217.9 ± 101.3 | 246.1 ± 70.3 |
| ***Reason for ICU admission [No (%) of patients]*** | |  |  |  |
| *Brain haemorrhage* | 14 (13.2) | 2 (22.2) | 6 (14.6) | - - |
| *Multiple injuries* | 17 (16.1) | 2 (22.2) | 9 (21.9) | - - |
| *Respiratory failure* | 47 (44.3) | 5 (55.5) | 13 (36.6) | 1 (33.3) |
| *Postoperative support* | 18 (16.9) | -- | 5 (12.2) | - - |
| *Other* | 10 (9.4) | -- | 7 (17.1) | 2 (66.7) |
| ***Mortality [No (%) of patients]*** | |  |  |  |
|  | 29 (27.4) | 2 (22.2) | 14 (34.1) | 0 (0) |

*^a^*pO_2_/FiO_2_ ratios as an index of the oxygenation. CPIS = clinical pulmonary infection score. SAPS = Simplified Acute Physiology Score, SD = standard deviation, TLR4 = Toll-like receptor 4, TIRAP = [TIR] – associated protein, VAP = ventilator associated pneumonia

**Table S5.** Detailed clinical characteristics of 54 matched patients (Group III) following elective cardiac surgery.

| ***Characteristic*** | ***Wild-type Control (n=18)*** | ***Any mutant TLR 4 (n=18)*** | ***TIRAP[hom] (n=5)*** | ***Any mutant TIRAP/TLR4 (n=13)*** |
| --- | --- | --- | --- | --- |
| *Age (years, mean ± SD)* | 68.5 ± 9.3 | 70.0 ± 9.7 | 67.8 ± 8.8 | 69.1 ± 9.2 |
| *Male/female* | 13/5 | 13/5 | 4/1 | 9/4 |
| *ASA class (mean ± SD)* | 3.3 ± 0.5 | 3.1 ± 0.3 | 3.2 ± 0.4 | 3.2 ± 0.4 |
| ***Procedures [No (%) of patients]*** | |  |  |  |
| *CABG* | 14 (77.8) | 15 (83.3) | 4 (80.0) | 12 (92.3) |
| *Valve repair* | 1 (5.6) | 0 (0.0) | 0 (0.0) | 0 (0.0) |
| *^a^Combined* | 2 (11.2) | 2 (11.2) | 1 (20.0) | 1 (7.7) |
| *^b^Other* | 1 (5.6) | 1 (5.6) | 0 (0.0) | 0 (0.0) |
| ***Mortality [No (%) of patients]*** | |  |  |  |
|  | 1 (5.6) | 0 (0.0) | 1 (20.0) | 1 (7.7) |

*^a^*Combined = CABG + Valve repair. *^b^*Other = Miscellaneous procedures on extracorporal circulation. ASA = American Society of Anesthesiologists, CABG = Coronary artery bypass grafting, SD = standard deviation.
